# Why is control of hypertension in sub-Saharan Africa poor?

**DOI:** 10.5830/CVJA-2015-065

**Published:** 2015

**Authors:** YK Seedat

**Affiliations:** Department of Internal Medicine, University of KwaZulu Natal, Durban, South Africa

**Keywords:** hypertension, control, sub-Saharan Africa

## Abstract

In sub-Saharan Africa (SSA) in 2010, hypertension (defined as systolic blood pressure ≥ 115 mmHg) was the leading cause of death, increasing 67% since 1990. It was also the sixth leading cause of disability, contributing more than 11 million adjusted life years. In SSA, stroke was the main outcome of uncontrolled hypertension. Poverty is the major underlying factor for hypertension and cardiovascular disease. This article analyses the causes of poor compliance in the treatment of hypertension in SSA and provides suggestions on the treatment of hypertension in a poverty-stricken continent.

## Abstract

In sub-Saharan Africa (SSA) in 2010, hypertension (defined as systolic blood pressure ≥ 115 mmHg) was the leading cause of death, increasing 67% since 1990. Hypertension was estimated to have caused over 500 000 deaths and 10 million years of life lost in 2010.^1^,^2^ It was also the sixth leading risk for a life of disability, contributing more than 11 million disability-adjusted life years.

Hypertension is the major cause of 50% of heart disease, stroke and heart failure. It is involved in 13% of deaths overall and over 40% of deaths in those with diabetes.^1^,^2^ Hypertension is a leading risk for foetal and maternal death during pregnancy, as well as for dementia and renal failure.^1^,^2^ In SSA, stroke, the major outcome of uncontrolled hypertension, has increased 46% since 1990 to become the fifth leading cause of death.^1^,^2^

In 1983, an age-adjusted prevalence study of the adult population of Durban showed that hypertension, according to the World Health Organisation (WHO) criteria, was highest in urban blacks of the Zulu tribe (25%), intermediate in whites (17%), lower in ethnic Indians, and lowest in rural blacks (9%). Our studies showed that 90% of our Zulu patients had undiagnosed hypertension, and 58% of Indian patients and 77% of white subjects had hypertension that was untreated or they had discontinued therapy.^3^ The first Demographic and Health Survey in South Africa in 1998 showed high levels of hypertension with inadequate treatment status.^4^

Low compliance is the main reason for poor control of blood pressure. Compliance has assumed great importance because it plays a major role in the successful treatment of health problems. Compliance is defined as the extent to which a person’s behaviour coincides with medical or health advice. This behaviour includes taking medication, keeping health-related appointments, and making lifestyle changes (diet, alcohol consumption, smoking cessation and physical exercise).

It is difficult to define compliance in terms of appointment keeping. Compliance is measured by quantitative and qualitative measurement of medications, pill counting, hospitalisation of patients, characteristics of medication, and physician–patient relationship. Physician compliance is assessed by patient perceptions, socio-demographic characteristics, behaviour modification, physician instruction, social support, and reduction in complexity of drugs.

Compliance in SSA is a major problem in the treatment of hypertension. However, little is known about the pricing of drugs in SSA. What is known is that the cost is borne by the patient (out-of-pocket expenditure) and medication is not subsidised by government or social insurance. Antihypertensive drugs within the same class and between classes have large differences in price. Those drugs listed in the WHO International Drug Indicator Guide were found to be cheaper. Adding advocated drugs onto countries’ national lists could reduce the price.

The Oxfam report^5^ (2007) titled ‘Pharma companies deny medicine to millions’ states that big pharmaceutical companies need to change the way they work, so as to reach 83% of the world’s consumers who don’t have access to medicines. The report lists the shortcomings of industry, which (1) had failed to implement a transparent, tiered pricing policy when prices are set for all essential medicines according to people’s ability to pay; (2) continues largely not to channel research and development into diseases that predominantly affect poor people in developing countries; (3) continues to be inflexible in protecting intellectual property, including challenging poor countries in court to stop using legal public health safeguards; and (4) continues to rely heavily on donations to get affordable medicines to people, even though this is unsustainable and sometimes counterproductive.

Affordability of drugs is defined as the number of days’ wages required for the lowest-paid individual to purchase a one-month supply of generic aspirin (100 mg), atenolol (100 mg), angiotensin converting enzyme inhibitor, lisinopril (10 mg) and simvastatin (20 mg) daily. The affordability of treatment for the secondary prevention of coronary heart disease would be 1.6 days in Bangladesh, 5.1 days in Brazil, 18.4 days in Malawi, 6.1 days in Nepal, 5.4 days in Pakistan and 1.5 days in Sri Lanka.^6^

In order to improve compliance in patients, drugs need to be made more affordable. Methods to improve affordability are: increase efficiency and volume of production of drugs, clarify treatment guidelines so that manufacturers can concentrate on fewer drugs, negotiate with manufacturers, publicise the lowest price, and reduce the credible threat of government action.^7^

Drugs are also not always equally available in SSA. The reasons for unavailability of drugs are: bureaucratic factors delay licensure and discourage manufacturers from introducing drugs into low-income countries, manufacturers’ prices are important causes of unaffordability, import tariffs, a lack of comparative price data, and mark-ups by distributers, pharmacies and dispensing doctors.

Poverty is the main underlying factor for hypertension and cardiovascular diseases.^8^ There are 57 countries in SSA. Africa has huge amounts of natural resources and is a source of natural strategic minerals. It is not overpopulated compared to the Asian continent yet economic conditions have deteriorated alarmingly in recent years. This is due to corruption and maladministration. Africa is the poorest continent and has the lowest per capita income in the world. The effect of poverty and its impact on health, particularly cardiovascular diseases, has been previously described.^8^

The number of poor people, defined as those making less than one dollar a day, has increased substantially in both relative and absolute terms. The absolute number of poor people has grown five times more than the figure for Latin America and twice that of South Asia. There is a critical shortage of doctors and nurse midwives to achieve 80% coverage for deliveries by skilled birth attendants, or for measles immunisation.

The staff shortages in SSA derive from a combination of underproduction of qualified healthcare workers, internal maladministration of professionals, and emigration of trained workers. The shortage of health workers in SSA reached 4.3 million in 2006, including 2.4 million doctors, nurses and midwives. It is more critical in rural areas as 38% of the world’s nurses and less than 25% of doctors work in rural areas.^9^

Obesity is associated with increased blood pressure levels in blacks. In the South African Demographic and Health Survey of 1998, the prevalence of obesity (body mass index ≥ 30 kg/m^2^ was 30% in black females and 8% in black males.4 It has been suggested that in Africa, the rural diet is relatively healthy, but with urbanisation, the diet is replaced with higher fat and lower carbohydrate intake. Poverty and cultural factors hinder the implementation of the DASH (Dietary Approach to Stop Hypertension) high-fruit, high-vegetable, low-salt diet, which was found to be very effective in African-Americans.^10^

For most low- and middle-income countries, the major obstacle to the control of blood pressure-related disease is the absence of appropriate primary healthcare services. Strategies for the prevention of cardiovascular disease (CVD) are to prevent the acquisition or enhancement of CVD risk factors. This is done by avoidance or decrease of the social, economic and cultural factors that contribute to the development of hypertension.

In primordial prevention, the main objective is to prevent the acquisition or enhancement of CVD risk factors. This is done by avoidance or decrease of the social, economic and cultural determinants that contribute to the development of hypertension. Primordial prevention relies on health policies that create a congenial environment to promote healthy behaviour, and population-wide education programmes. These in turn depend on many factors, including the involvement of political leaders and the mass media. Primordial prevention relies on healthy behaviour and population-wide education programmes.

Primary prevention is to reduce or reverse the risk factors in urban communities. This is done by reducing the risk factors for hypertension through appropriate policies, or modifying the risk factors in order to prevent or delay the development of hypertension. Since a substantial number of adults have blood pressure above the optimal level, even a small reduction in blood pressure can produce a significant decrease in cardiovascular risk in the population. At the individual level, primary prevention of hypertension consists of adopting healthy lifestyles at an early age. These should be non-pharmacological, populationbased and lifestyle-linked measures, such as salt restriction in food, increased exercise, control of obesity, increased potassium consumption in the form of fresh fruit and vegetables, reduced alcohol intake, and stopping cigarette smoking.

There is a need to develop cost-effective methods for the diagnosis of hypertension, and low-cost, saving measures such as microscopic urine examination, and testing for urinary albumin and glucose levels, and serum creatinine, potassium and glucose levels. Coronary heart disease, while rising in incidence, is still relatively uncommon in SSA. Because serum cholesterol levels in blacks are lower than in whites and the Indian population in SSA, it may not be necessary to routinely measure lipid patterns in black hypertensive patients.^10^ However, we need data on the cost effectiveness of routine measuring of lipid patterns in blacks, with the current stage of knowledge of elevation of serum lipid levels in many individuals.

At a tertiary level, we need to avoid high-cost, low-yield technologies such as routine echocardiography for hypertension, and computerised resonance tomography and magnetic tomography for reno-vascular hypertension.^8^ While it is important to use modern technology in medicine for the treatment of hypertension, particular attention should be given to cost-effectiveness and affordability, as many countries in SSA have severe resource constraints.^9^-^11^ In some countries, the health budget per capita does not exceed US$10 per year and this is completely insufficient to address the needs created by the double burden of non-communicable and infectious diseases, such as HIV/AIDS.

For most low- and middle-income countries in SSA, the major obstacle is the absence of appropriate healthcare services. In many regions, primary care provides only episodic care with little record kept of previous visits. These services must be adapted not only for the management of blood pressure-related disease but also for the management of other serious diseases, including HIV infection. Many of the clinics are poorly staffed and lack adequate equipment and medication. It is the norm to have large queues of patients waiting for many hours after entry to a clinic, to be seen by nursing or medical personnel.^12^

## Conclusion

Treatment of hypertension in the poverty-stricken continent of SSA presents a challenge that needs to be addressed urgently. A similar plea has been made to the South African medical fraternity.^13^ The future approach to CVD prevention should be aimed at societal change throughout the life of the individual, and on large changes in multiple risk factors. There is a need to solve the problem by working with goverments, the WHO and other national and international organisations.

We must remember the wise words of our beloved Nelson Mandela, ‘We must face the matter squarely, that where there is something wrong in how we govern ourselves, it must be said that the fault is not in the stars, but in ourselves. We know that we have it in ourselves as Africans to change all this. We must assert our will to do so; we must say there is no obstacle (large) enough to stop us bringing about an African renaissance’.
